# Supplementary material for: T-Lymphocyte Phenotypic and Mitochondrial Parameters as Markers of Incomplete Immune Restoration in People Living with HIV+ on Long-Term cART
Source: Biomedicines. 2025 Jul 28;13(8):1839. doi: 10.3390/biomedicines13081839 (PMC12383652; doi:10.3390/biomedicines13081839)
Supplement: Supplementary file 1 [file biomedicines-13-01839-s001.zip › biomedicines-3722820-supplementary.pdf]

Supplementary Table S1. Phenotypic markers and subsets of CD4 and CD8 T cell pools in the studied groups. P[1] ART+ vs ART-; P[2] ART+ vs HC; p3 ART- vs HC

		ART+	ART-	HC	P1 ART+ vs ART-	P2 ART+ vs HC;	p3 ART- vs HC
**T CD8+ CM**	**% median (IQR)**	**12.7(6.3-18.4)**	**2.9(1-4.9)**	**8.7(5.2-13.9)**	********	**ns**	********
**T CD8+ N**	**% median (IQR)**	**23.6(17.4-29.8)**	**8.8*4-14.2)**	**29.3(18.8-39.7)**	*******	**ns**	********
**T CD8+ EM**	**% median (IQR)**	**34.3(27.6-46.3)**	**58(47.5-70.2)**	**33.6(26.6(44.6)**	********	**ns**	********
**T CD8+ TE**	**% median (IQR)**	**25.1(17.1)**	**24(15.2-34.8)**	**19.6(11-30.4)**	**ns**	**ns**	**ns**
**T CD8 PD-1**	**GeoMean**	**277(226-303)**	**320(285-375)**	**210(175-272.5)**	*****	**ns**	********
**T CD4 CM**	**% median (IQR)**	**35.9(31.7-47.6)**	**25.1(16.2-33.3)**	**39.4(32.8-44.1)**	*******	**ns**	*******
**T CD4 N**	**% median (IQR)**	**34.4(24.7-41.5)**	**34.8(13.1-46.4)**	**31.4(23.2-40.8)**	**ns**	**ns**	**ns**
**T CD4 EM**	**% median (IQR)**	**17.5(13.7-24.6)**	**27(16-37.6+)**	**22.8(18.3-30.1)**	*****	**ns**	**ns**
**T CD4 TE**	**% median (IQR)**	**5.3(2.1-8.8)**	**7.2(4.5-18.5)**	**3.2(1.6-4.4)**	**ns**	*****	********
**T CD4 PD-1**	**GeoMean**	**273(213-328)**	**329(263-403)**	**267(230.3-370)**	**ns**	**ns**	**ns**
**T CD8 CD27+CD28+**	**% median (IQR)**	**53.2(37.7-61.6)**	**22.6(16.3-33.1)**	**61.8 (53.8-84.33)**	********	**ns**	********
**CD25+CD127- Treg**	**% median (IQR)**	**3.35(2.55-4.73)**	**4.9(2.6-6.40**	**2.7(1.8-3.6)**	**ns**	**ns**	******
**CD39+**	**% median (IQR)**	**13.65(3.23-33.93)**	**20.7(8.45-41.85)**	**18.1(5.6-31.6)**	**ns**	**ns**	**ns**
**CD39+**	**GeoMean**	**956(806-1341)**	**1230(755-1577)**	**1351(958-1548)**	**ns**	**ns**	**ns**
**CD38+ CD4+**	**% median (IQR)**	**45.1(39-59.5)**	**72.4(51.6-80)**	**43.7(35.1-53)**	*******	**ns**	********
**CD38+ CD8+**	**% median (IQR)**	**27.9(18.9-41.6)**	**79(58.2-91)**	**25.1(11.7-39)**	********	**ns**	********
**CD8+CD38+**	**Molecules**	**836(666-1174)**	**3711(1711-5947)**	**749 (626-865)**	********	**ns**	********
**CD4+CD38+**	**Molecules**	**1291(1002-1957)**	**2474(1552-4202)**	**1021 (873-1379)**	********	**ns**	********
**CD8+ CD27-CD28-**	**% median (IQR)**	**34.8 (28.5-55)**	**57.8 (39.1-67)**	**24.75 (8.67-33.5)**	*****	*****	********

CD8+ CD57-CD28+	% median (IQR)	53.3 (39.7-61.9)	24.5(19.2-34.4)	61 (50.53-84.23)	****	ns	****
CD8+ CD57+CD28+	% median (IQR)	1.9 (0.6-3.13)	1 (0.7-1.5)	2.25 (0.95-3.55)	ns	ns	ns
CD8+ CD57+CD28-	% median (IQR)	30.4 (19.1-45.3)	35.4 (21.3-42.5)	19.4 (6.2-37.88)	ns	ns	ns
CD8+ CD57-CD28-	% median (IQR)	10.3 (6.7-14.6)	38.4 (28.2-48.1)	7.8 (5.43-11)	****	ns	****
CD8+ CD57-CD27+	% median (IQR)	54.6(36.5-62.8)	32.6(24.2-51.1)	63.9(56.3-82.1)	*	*	****
CD8+ CD27+CD28-	% median (IQR)	5.15(3.2-9.1)	18.2(12.6-27.7)	5.3(3.05-10.68)	****	ns	***
CD8+ CD28+ CD27-	% median (IQR)	3.4 (2.4-5.1)	2.3 (1.3-3)	1.7 (1.1-5.65)	*	ns	ns
CD8+ CD57+TIGIT-	% median (IQR)	9.2 (4.3-21.8)	12.5 (3.3-16.8)	3 (1.5-7.30	ns	***	**
CD8+ CD57+TIGIT +	% median (IQR)	19.4(12.3-29.9)	22.3(12.1-30.9)	16.4(9-29.2)	ns	ns	ns
CD8+ CD57-TIGIT+	% median (IQR)	26(18.6-36.8)	44.7(34-51.8)	24.3(18.4-32.8)	****	ns	****
CD8+ CD57- TIGIT -	% median (IQR)	34.8(25-45.9)	20.1(11.3-28.9)	50.3(33.5-67.3)	***	ns	****
CD8+ CD57-CD27-	% median (IQR)	9.9(6.9-13.3)	22.4(17.5-34.1)	6.6(4.6-14.2)	****	ns	****
CD8+ CD27+ TIGIT -	% median (IQR)	31.3(22.7-43.2)	14.7(8.5-23.2)	41.5(31.9-60.5)	****	ns	****
CD8+ CD27+ TIGIT+	% median (IQR)	23.5 (16.3-29.80)	28.1 (22-38.5)	27.2 (18.3-34)	ns	ns	ns
CD8+ TIGIT+	% median (IQR)	52.9 (42.5-60.6)	66.9 (55.3-79.1)	41.1 (32.4-56.2)	**	ns	****
CD8+ CD27+CD28- TIGIT+CD57-	% median (IQR)	53.9 (45.5-68.9)	74.1 (60-80.1)	40.1 (26.7-58.5)	**	*	****
CD8+ CD27+CD28- TIGIT+CD57+	% median (IQR)	25.9 (15.6-36.3)	13.65 (7.23-26.53)	28.3 (19-49.9)	ns	ns	**
CD8+ CD27+CD28- TIGIT-CD57-	% median (IQR)	9.9 (6.4-17.1)	7.8 (3.2-10.5)	14.55 (3.58-24.88)	ns	ns	ns
CD8+ CD27+CD28- TIGIT-CD57+	% median (IQR)	3.6 (1.7-7.5)	2.5 (1.4-5.8)	2.65 (1.33-7.43)	ns	ns	ns
CD8+ CD27+CD28+ TIGIT+CD57-	% median (IQR)	35.6 (24.1-44.6)	43.5 (36.7-52.8)	24.5 (17.6-46.2)	*	ns	**

**CD8+ CD27+CD28+ TIGIT+CD57+**	**% median (IQR)**	**2.5 (0.9-4.9)**	**2.3 (1.5-7.5)**	**2.1 (1.23-5.13)**	**ns**	**ns**	**ns**
**CD8+ CD27+CD28+ TIGIT-CD57-**	**% median (IQR)**	**59.6 (51-72.4)**	**49 (38.2-60.9)**	**73.25 (49.35-80.30**	*****	**ns**	******
**CD8+ CD27+CD28+ TIGIT-CD57+**	**% median (IQR)**	**0.1 (0.0-0.2)**	**0.1 (0.0-0.4)**	**0.1 (0.0-0.4)**	**ns**	**ns**	**ns**
**CD8+ CD27-CD28- TIGIT+CD57-**	**% median (IQR)**	**12.8 (9.4-21.2)**	**30.1 (20.9-41.1)**	**13.4 (9.2-26.7)**	*******	**ns**	******
**CD8+ CD27-CD28- TIGIT+CD57+**	**% median (IQR)**	**46.1 (28.6-60.3)**	**30.9 (21.7-42.8)**	**44.9 (36.9-68.5)**	*****	**ns**	*****
**CD8+ CD27-CD28- TIGIT-CD57-**	**% median (IQR)**	**3.4 (2.3-5.1)**	**7.6 (2.8-16.6)**	**4.8 (2.4-11.7)**	*****	**ns**	**ns**
**CD8+ CD27-CD28- TIGIT-CD57+**	**% median (IQR)**	**28.8 (16.2-54.9)**	**25.4 (8.5-30.9)**	**18.3 (6.9-36.5)**	**ns**	**ns**	**ns**
**CD8+ CD27-CD28+ TIGIT+CD57-**	**% median (IQR)**	**49.3 (39-59.7)**	**48.9 (34.9-62.6)**	**41 (27.1-57)**	**ns**	**ns**	**ns**
**CD8+ CD27-CD28+ TIGIT+CD57+**	**% median (IQR)**	**5.5 (3.6-11.4)**	**5 (1.8-11.7)**	**5.7 (0.0-13.18)**	**ns**	**ns**	**ns**
**CD8+ CD27-CD28+ TIGIT-CD57-**	**% median (IQR)**	**34.2 (25-52.1)**	**42.9 (23.8-54.1)**	**49.7 (30-64.8)**	**ns**	**ns**	**ns**
**CD8+ CD27-CD28+ TIGIT-CD57+**	**% median (IQR)**	**1.9 (0.0-5.6)**	**1.7 (0.0-4.8)**	**1.5 (0.0-3.53)**	**ns**	**ns**	**ns**
**CD4+ CD57+TIGIT-**	**% median (IQR)**	**1.8(0.9-3.7)**	**2.4(1.53-5.83)**	**1.9(08-4.8)**	**ns**	**ns**	**ns**
**CD4+ CD57-TIGIT-**	**% median (IQR)**	**70(67.1-76.7)**	**58(49.2-68)**	**75.2(64.3-80.3)**	******	**ns**	*******
**CD4+ CD57+TIGIT+**	**% median (IQR)**	**2.4(1.1-4.1)**	**4.9(3.5-11)**	**2(1.1-4.5)**	*******	**ns**	*******
**CD4+ CD57-TIGIT+**	**% median (IQR)**	**23(17.5-27.2)**	**25.7(20.1-38)**	**19.8(15.7-23.1)**	**ns**	**ns**	******
**CD4+ CD27+TIGIT-**	**% median (IQR)**	**63 (58.5-71)**	**46.6 (32.8-61.5)**	**61.6 (56-70.2)**	*******	**ns**	******
**CD4+ CD27+TIGIT+**	**% median (IQR)**	**19 (14.6-26.2)**	**15.2 (13.3-23.9)**	**16.8 (14.4-22.3)**	**ns**	**ns**	**ns**

**CD4+ CD27-TIGIT-**	**% median (IQR)**	**8.8 (6.2-13.4)**	**11.9 (5.9-22.9)**	**13.4 (7.4-18.9)**	**ns**	**ns**	**ns**
**CD4+ CD27-TIGIT+**	**% median (IQR)**	**4.7 (2.8-10.3)**	**14 (8.3-25.8)**	**5.1 (2.6-7.3)**	*******	**ns**	********
**CD4+ CD27+CD28-**	**% median (IQR)**	**0.9 (0.6-1.3)**	**2.1 (1.1-2.9)**	**1.3 (0.88-1.83)**	********	**ns**	**ns**
**CD4+ CD27+CD28+**	**% median (IQR)**	**81.3 (72.8-86.5)**	**61.3 (45.5-74.6)**	**82.15 (72.25-90.33)**	********	**ns**	*******
**CD4+ CD27-CD28-**	**% median (IQR)**	**7.9 (3.5-17.2)**	**21.8 (14.4-41.4)**	**9.45 (2.22-17.28)**	********	**ns**	*******
**CD4+ TIGIT+**	**% median (IQR)**	**26.1 (18.7-30.5)**	**35.7 (27.6-42.5)**	**21.7 (17.4-27.2)**	*****	**ns**	*******
**CD4+ CD27+CD28-TIGIT+CD57-**	**% median (IQR)**	**39.1 (30.8-60)**	**54.4 (37.5-66.9)**	**41.3 (23.8-50.8)**	**ns**	**ns**	**ns**
**CD4+ CD27+CD28-TIGIT+CD57+**	**% median (IQR)**	**10.3 (4.5-16.7)**	**9.9 (1.9-14.1)**	**10.5 (6-20.1)**	**ns**	**ns**	**ns**
**CD4+ CD27+CD28-TIGIT-CD57-**	**% median (IQR)**	**48.7 (23.5-60)**	**33.3 (20-41.7)**	**36.5 (20.6-54.3)**	**ns**	**ns**	**ns**
**CD4+ CD27+CD28-TIGIT-CD57+**	**% median (IQR)**	**0.0 (0.0-1.7)**	**0.0 (0.0-1.2)**	**4 (0.0-11.2)**	**ns**	**ns**	**ns**
**CD4+ CD27+CD28+TIGIT+CD57-**	**% median (IQR)**	**22.5 (16.9-26.4)**	**28.1 (17.8-39.6)**	**20.1 (14-23.7)**	**ns**	**ns**	******
**CD4+ CD27+CD28+TIGIT+CD57+**	**% median (IQR)**	**0.3 (0.1-0.5)**	**0.4 (0.1-0.7)**	**0.35 (0.1-0.5)**	**ns**	**ns**	**ns**
**CD4+ CD27+CD28+TIGIT-CD57-**	**% median (IQR)**	**77.1 (72.7-82.6)**	**71.5 (59.5-82)**	**79.6 (75.93-86.1)**	**ns**	**ns**	******
**CD4+ CD27+CD28+TIGIT-CD57+**	**% median (IQR)**	**0.1 (0.0-0.2)**	**0.1 (0.0-0.2)**	**0.1 (0.0-0.2)**	**ns**	**ns**	**ns**
**CD4+ CD27-CD28-TIGIT+CD57-**	**% median (IQR)**	**22.5 (13.9-35.9)**	**33.3 (17.5-44.9)**	**16 (8.5-26.4)**	**ns**	**ns**	*****
**CD4+ CD27-CD28-TIGIT+CD57+**	**% median (IQR)**	**19.7 (15-28.7)**	**15.6 (13.3-26.7)**	**32.3 (13.5-55.9)**	**ns**	**ns**	**ns**
**CD4+ CD27-CD28-TIGIT-CD57-**	**% median (IQR)**	**21.9 (11.2-34.1)**	**24.1 (7-45.3)**	**8.6 (5.0-40.9)**	**ns**	**ns**	**ns**

**CD4+ CD27-CD28- TIGIT-CD57+**	**% median (IQR)**	**23.6 (10.3-40.4)**	**13.5 (6.3-24.6)**	**15.5 (11.3-42.2)**	**ns**	**ns**	**ns**
**CD4+ CD27-CD28+ TIGIT+CD57-**	**% median (IQR)**	**20 (14.3-27.7)**	**27.8 (14.7-38.9)**	**11.9 (7.2-25.8)**	**ns**	**ns**	******
**CD4+ CD27-CD28+ TIGIT+CD57+**	**% median (IQR)**	**0.5 (0.0-1.3)**	**0.3 (0.0-2.4)**	**0.6 (0.0-1.5)**	**ns**	**ns**	**ns**
**CD4+ CD27-CD28+ TIGIT-CD57-**	**% median (IQR)**	**76.3 (70.3-84.1)**	**68.9 (58.1-83.6)**	**84.3 (73.4-91.1)**	**ns**	**ns**	******
**CD4+ CD27-CD28+ TIGIT-CD57+**	**% median (IQR)**	**0.8 (0.3-1.9)**	**1.5 (0.6-3.4)**	**0.6 (0.0-2.8)**	**ns**	**ns**	**ns**
